# Food consumption score and the Nexus of maternal nutritional status among pregnant women in Gamo Zone, South Ethiopia

**DOI:** 10.3389/fnut.2025.1498599

**Published:** 2025-04-24

**Authors:** Teshale Fikadu, Dessalegn Tamiru, Beyene Wondafrash Ademe

**Affiliations:** ^1^School of Public Health, College of Medicine and Health Science, Arba Minch University, Arba Minch, Ethiopia; ^2^Department of Nutrition and Dietetics, Institute of Health, Jimma University, Jimma, Ethiopia

**Keywords:** associated factors, food consumption score, nutritional status, pregnant women, South Ethiopia

## Abstract

**Background:**

Food consumption score is an indicator used to evaluate food security, ensuring that individuals have reliable access to sufficient, safe, and nutritious food that meets their dietary needs and preferences for an active and healthy life. Poor nutrition during the first 1,000 days of life can result in developmental delays, stunted growth, cognitive impairments, and a higher risk of chronic diseases later in life. This study, therefore, aims to evaluate the level of food consumption scores and its associated factor among pregnant women.

**Methods:**

A community-based cross-sectional study was conducted among 638 randomly selected pregnant women from October to March 2023 at the Arba Minch Health and Demographic Surveillance site in Southern Ethiopia. Data were collected using a pre-tested, interviewer-administered structured questionnaire. Modified Poisson regression was employed to measure prevalence ratios, while both bivariate and multivariable analyses were performed to identify potential variables for further analysis and to determine factors associated with an acceptable food consumption score, respectively.

**Result:**

Among the 638 pregnant women, 8.15% (95% CI: 6.26, 10.55) had poor, 14.89% (95% CI: 12.33, 17.87) had borderline, and 76.96% (95% CI: 73.52, 80.07) had acceptable food consumption scores While 31.97% of the pregnant women were suffering from under nutrition. Acceptable food consumption score was higher among women who were urban dwellers (APR = 1.09; 95% CI: 1.02, 1.20), in higher economic status (APR = 1.05; 95% CI: 1.01, 1.16), had planned pregnancy (APR = 1.13; 95% CI: 1.02, 1.25), were exposed to mass media (APR = 1.19; 95% CI: 1.07, 1.31), had a vegetable garden (APR = 1.14; 95% CI: 1.04, 1.25), attend health facility for antenatal care (APR = 1.13; 95% CI: 1.02, 1.26), and those who consumed food four or more times per day (APR = 1.23; 95% CI: 1.11, 1.36). Also the level of acceptable food consumption score increased by 2, 3, and 4% for every one-unit increase in BMI (APR = 1.02; 95% CI: 1.01, 1.04), MUAC in cm (APR = 1.03; 95% CI: 1.01, 1.05), and gestational age in weeks (APR = 1.04; 95% CI: 1.01, 1.07), respectively.

**Conclusion:**

Nearly one-third and three-fourths of the pregnant women were undernourished and had acceptable food consumption scores, respectively. Factors associated with acceptable food consumption scores included place of residence, wealth status, planned pregnancy, exposure to mass media, having a vegetable garden, attending antenatal care at health facilities, consuming four or more meals daily, and gestational age. Thus addressing these factors is crucial for improving FCS among pregnant women. Furthermore, efforts should be directed toward increasing media exposure, boosting household income, promoting vegetable gardening, and improving planned pregnancies and antenatal care visits.

## Introduction

Maternal nutrition represents a significant public health challenge as it influences the health of the mother, the fetus, and subsequent generations (1). Meeting the nutritional demands for fetal growth and maternal tissue expansion requires additional nutrients (2). However, pregnant women in low and middle-income countries like Ethiopia typically consume diets based on a limited number of staple foods (primarily cereals), resulting in gaps between their dietary intake and the nutritional requirements for various nutrients (2, 3).

The first 1,000 days of human life are a crucial period not only for growth and development but also for long-term health impacts (4). Evidence indicates that inadequate nutrition during this period can cause developmental delays, stunted growth, cognitive impairments, and increased susceptibility to chronic diseases in adulthood (5).

Adequate and quality dietary intake is crucial for pregnant women to meet nutritional requirements for anatomical, physiological, and biochemical changes during pregnancy (6). Also it plays a pivotal role in enhancing pregnancy outcomes, preserving maternal health, and promoting fetal growth and development (7, 8). However, suboptimal intake increases the risk of maternal morbidity, intrauterine growth retardation (IUGR), prematurity, stillbirth, low birth weight (LBW), maternal and prenatal death and reduces gestational weight gain (8–11).

In developing countries like Ethiopia, dietary practices during pregnancy are shaped by restrictions on essential foods and/or drinks considered unacceptable in society, rooted in religious, cultural, historical, and social norms (12, 13). A recent systematic review in Ethiopia found that around 34.22% of pregnant women are restricted from consuming essential foods due to cultural taboos, with the prevalence varying significantly by region. The variation in food taboos across regions, with 67.4% in the Somali region compared to 11.5% in Tigray region, highlights significant socio-cultural differences that influence maternal nutrition (14). The most commonly prohibited foods include cereals (such as wheat, maize, and millet), nuts (groundnuts), vegetables (like green pepper, spinach, kale, broccoli, and cabbage), fruits (banana and tomato), and animal source foods (such as meat, dairy, and eggs) (14, 15). This restriction significantly impacts the FCS, even among households considered food-secure, consequently increasing the risk of malnutrition.

The prevalence of under nutrition among pregnant women in Ethiopia varies significantly, ranging from 21.8 to 43.1% (16–18); with certain regions showing even higher rates. Similarly, the level of adequate dietary practices during pregnancy is generally low and varies across the country, with Addis Ababa having the highest at 63.9% and eastern Ethiopia the lowest at 15.2% (19, 20). Furthermore, maternal dietary knowledge during pregnancy is also insufficient, with the highest proportion of good dietary knowledge in Addis Ababa at 73.9% and the lowest in southwest Ethiopia at 21% (19, 21).

The FCS is a food frequency indicator developed by the World Food Programme (WFP) aiming to capture both the quantity and quality of dietary intake (22). It serves as an indicator for the diversity index, balanced food consumption, and composite score of dietary diversity, food frequency, and the relative nutritional significance of various food groups (23, 24). It is among the indicators used to assess food security, which ensures that every person has consistent access to sufficient safe and nutritious food meeting their dietary needs and preferences, enabling them to lead an active and healthy life (25).

In southern Ethiopia, the level of household food insecurity was 50, and 54% of pregnant women lived in food-insecure households (26, 27), which is significantly associated with under nutrition during pregnancy (28). Additionally, the FCS ranged from 57.6 to 68.3% for acceptable, 17.4 to 35.2% for borderline, and 7.1 to 14.3% for poor, indicating that household-level FCS was low (25, 29). Furthermore, 36.4% of households experienced both poor dietary diversity and a lack of stable access to, as well as availability of, sufficient food (25), suggesting that both diversified food consumption and access to food were low. All of these factors contribute to under nutrition, which is particularly severe during pregnancy when nutritional requirements are high.

The FCS among pregnant women in Ethiopian ranges from 51.20 to 81.50% acceptable, 16.60 to 42.60% borderline, and 1.90 to 10.49% poor (30–32). Factors influencing acceptable FCS included socioeconomic status (30, 32), residence (31), religion (31), maternal educational status (32), ANC follow-up (30), consumption of animal source foods (30), possession of agricultural land (30), skipping meal (32), and attitudes toward dietary diversity during pregnancy (30, 32). However, it did not address the association between nutritional status and FCS (30, 31). In addition, dietary practices were significantly influenced by geographical and sociocultural variations, underscoring the necessity for tailored interventions. Hence, this study aims to evaluate the factors influencing FCS and its association with nutritional status among pregnant women in Gamo Zone, South Ethiopia.

## Materials and methods

### Study setting, design, and period

A study involving 638 randomly selected pregnant women was conducted in Arba Minch Zurea and Gacho Baba districts of southern Ethiopia from October to March 2023. This study aimed to establish a baseline for a prospective cohort investigating the relationship between dietary practices and newborn nutritional status.

The provincial capitals, Arba Minch Zurea and Gacho Baba, are located 437 km and 439 km south of Addis Ababa, the capital of Ethiopia, respectively. The two districts have a total population of 164,629 (82,499 male and 82,630 female) as of the year 2023/24, projected from the 2007 CSA census data. Among them, 74,257 reside in the Arba Minch Health and Demographic Surveillance site (AM-HDSS), which covers 9 representative kebeles (the smallest administrative unit in Ethiopia) from these districts. In the surveillance site, it was anticipated that 2,598 women would be pregnant for the year 2023/24 and would access healthcare from 7 nearby health centers, 37 health posts, and private healthcare facilities.

### Source population and study population

All pregnant women identified in AM-HDSS constituted the source population, while the study population comprised selected pregnant women from AM-HDSS who met the inclusion criteria.

### Inclusion and exclusion criteria

All pregnant women who were permanent residents of AM-HDSS were included in the study, except for those who were critically ill, declined to participate, or had known chronic conditions like HIV and diabetes mellitus.

### Sample size determination

Single population proportion formula was used to calculate the sample size, assuming a 95% confidence interval, 5% precision level, and an expected prevalence of acceptable food consumption score of 54.46%, as reported in a study from Eastern Ethiopia (30), with a 10% possible non-response.


$$n=\;\frac{{\left(Z_{1-\alpha /2}\right)}^{2}*P\left(1-P\right)}{{\left(d\right)}^{2}}\;n=\;\frac{{\left(1.96\right)}^{2}*0.5446\left(1-0.5446\right)}{0.05^{2}}=381$$

Where

n = Desired number of samples.

P = expected Proportion of acceptable food consumption score = 54.46%.

Z_1-a/2_ = Standard value for 95% CI = 1.96.

d = 5%.

The calculated sample size was 419; however, the baseline survey of the prospective cohort study assessed 638 pregnant women. Therefore, all 638 participants were included in this analysis.

### Sampling techniques

The sample was allocated proportionally to each kebele based on the anticipated number of pregnant women, and participants were selected using a computer-generated simple random sampling method.

### Data collection tools, procedures, and measurements

A structured questionnaire, pre-tested and administered by face-to-face interviewers, was used to collect data on maternal socio-demographic, health and dietary factors. Dietary data was collected using a validated food frequency questionnaire (FFQ) (33) customized for local conditions. This FFQ included recalling food intake over 24 h and dietary habits over one week, covering a total of 46 food items categorized into ten groups to assess both actual and habitual dietary intake, respectively.

The data were digitally collected with the help of an open-source toolkit (Kobo Collect). This mobile app is user-friendly and functions offline. A questionnaire template was developed in a computer database, uploaded to a server, and then downloaded to the data collectors’ mobile phones via an internet connection. Specific training was provided on how to download the template and upload data (34).

Maternal nutritional status was assessed using mid-upper arm circumference (MUAC) and body mass index (BMI). MUAC was measured using a flexible, non-stretchable standard tape, placed at the midpoint between the acromion process and olecranon, with an accuracy of 0.1 cm (17). Weight was assessed in kilograms (kg) using a WHO standard scale accurate to 0.1 kg, with participants wearing light clothing. Height was measured standing upright without shoes, using a stadiometer accurate to 0.1 cm. then, BMI was computed by dividing body weight in kilograms by the square of height in meters (35).

Maternal dietary knowledge was evaluated using questions adapted from previous research. Responses were categorized as either “0” for incorrect answers indicating lack of awareness, or “1” for correct answers indicating awareness. Scores ranged from 0 to 100%. Mothers scoring below 60% were categorized as having “poor knowledge,” those scoring between 60 and 79% were categorized as having “moderate knowledge,” and those scoring 80% or higher were categorized as having “good knowledge” (36–38).

Household wealth status was assessed using items adapted from the 2016 EDHS. These items included the number of livestock owned, availability of agricultural land, materials used in house construction, source of drinking water, presence of electricity, type of cooking fuel used, and ownership of modern household goods (e.g., bicycle, television, radio, motorcycle, sewing machine, telephone, car, refrigerator, mattress, bed, and mobile phone). The assumption was that owning these assets, services, and amenities reflects the economic standing of the household (39).

The meal frequency of pregnant women was assessed based on the number of meals (main and snacks) consumed per day. Those consuming more than three meals were categorized as having adequate meal frequency, while those consuming three or fewer meals were categorized as having inadequate meal frequency (18).

Minimum Dietary Diversity for Women (MDDW) was assessed, with pregnant women classified as having adequate minimum diet diversity if they consumed at least 5 of the 10 food groups in the past 24 h (40).

Animal source food consumption (ASFC) among pregnant women was estimated by adding up all animal source foods consumed over the previous 7 days and converting the total into terciles. Pregnant mothers in the highest tercile were categorized as having “high” ASFC, while those in the lower terciles were categorized as having “low” consumption (30).

The Food Variety Score (FVS) is determined by summing all the foods pregnant women have eaten in the last 7 days and the mean was computed. Pregnant women with FVS higher than mean score classified as having a “high,” otherwise classified as having a “low” food variety score (30).

The FCS is a sensitive measure of food frequency and dietary diversity, evaluated using WFP guidelines based on the previous 7 days of survey data. Each food item receives a score from 0 to 7, depending on how many days it was consumed. These items are categorized into groups, and the frequencies of all items within each group are added together. The FCS is computed by multiplying the frequency of each food group by its respective weight, and then summing these values to create a composite score. This score is classified as follows: Poor food consumption score ranges from 0 to 28, Borderline food consumption score ranges from 28.5 to 42, and Acceptable food consumption score is greater than 42 (41).

### Data quality control

Proper data collection tools were developed and utilized to ensure data quality. Trained data collectors were regularly supervised to guarantee accurate data collection. All collected data were submitted daily, checked for completeness and consistency, and approved by supervisors. Following a pre-test among 35 pregnant women in the non-AM-HDSS kebele, the questionnaire was revised, reducing the number of food items from 50 to 46.

### Data processing and analysis

All collected and submitted data were downloaded, cleaned in Excel, exported to, and analyzed with STATA 16.0 version statistical software. Two-way tables with Pearson’s chi-square test or Fisher’s exact test were used to present and compare food consumption scores for categorical predictors, while means with standard deviations were used for continuous variables. Exploratory factor analysis using the principal component analysis (PCA) method was employed to determine household wealth status and maternal dietary knowledge. Sample adequacy, Bartlett’s test for sphericity, commonality tests, and assessments for lack of correlation assumptions in complex structures were conducted. PCA identified four factors explaining 67 and 75% of the total variance, respectively. The factor value of the first component, explaining the maximum variance, was used and ranked into terciles to classify household wealth status and maternal dietary knowledge.

Modified Poisson regression model was used to improve accuracy and robustness while directly measuring prevalence ratios (PRs). Variables with *p*-values ≤0.25 from bivariate analysis, were entered into a multivariable regression model to identify the determinants of acceptable food consumption scores. Adjusted prevalence ratios (APRs) with 95% confidence intervals were used to determine the direction and strength of associations. Statistical significance was defined as a *p*-value less than 0.05. The deviance, Akaike’s information criterion (AIC) and Bayesian information criterion (BIC) test were used to assess the model fitness, and multicollinearity test was used to determine correlations between independent variables using variance inflation coefficients (VIF).

## Results

### Baseline characteristics of the study participant

A total of 638 pregnant women participated in the study, with a mean age of 26.29 years (± 5.77), ranging from 17 to 42 years, at 16 to 20 weeks of gestation. The majorities of the respondents attended formal education (62.85%), were rural dwellers (90.28%), were aged between 20 to 35 years (80.56%), were housewives (67.08%) visited health facilities for ANC services (67.87%), and had planned pregnancies (67.87%). Almost one-third of the respondents reported no exposure to media and did not have a vegetable garden (Table 1).

**Table 1:** Baseline characteristics of the study participants in AM-HDSS, Gamo Zone of South Ethiopia.

Variable	Category	Number	Percent
Maternal age	Less than 20 years	81	12.70
	20 to 35 years	514	80.56
	36 years and more	43	6.74
	Mean (± SD)	26.29 (± 5.77)	
Place of residence	Rural	576	90.28
	Urban	62	9.72
Religion	Orthodox	123	19.28
	Protestant	515	80.72
Educational status	No formal education	237	37.15
	Attend formal education	401	62.85
Partner educational status	Unable to Read and write	273	42.79
	Read and write only	365	57.21
Occupation	House wife	428	67.08
	Farmer	82	12.85
	Merchant	74	11.60
	Government employ	34	5.33
	Student	20	3.13
House hold asset decision making	By both partner	118	18.50
	Mainly by husband	520	81.50
Number of family member	Less than five	309	48.43
	Five and more	329	51.57
	Mean (± SD)	4.30 (1.78)	
Wealth status	Low	218	34.17
	Middle	208	32.60
	High	212	33.23
Birth interval	less than 2 years	59	17.61
	2 years and more	276	82.39
Pregnancy status	Planned	433	67.87
	Unplanned	205	32.13
ANC visit	Yes	433	67.87
	No	205	32.13
Had nausea vomiting	Yes	339	53.13
	No	299	46.87
Had obstetric complication	Yes	11	1.72
	No	627	98.28
Media exposure	Yes	432	67.71
	No	206	32.29
Had vegetable garden	Yes	409	64.11
	No	229	35.89
Number of pregnancy	Mean (± SD)	3.02 (± 1.70)	
Number of delivery	Mean (± SD)	2.50 (± 1.46)	
Gestational age	Mean (± SD)	18.17 (± 1.72)	

### Food consumption score and nutritional status

**Figure 1:**
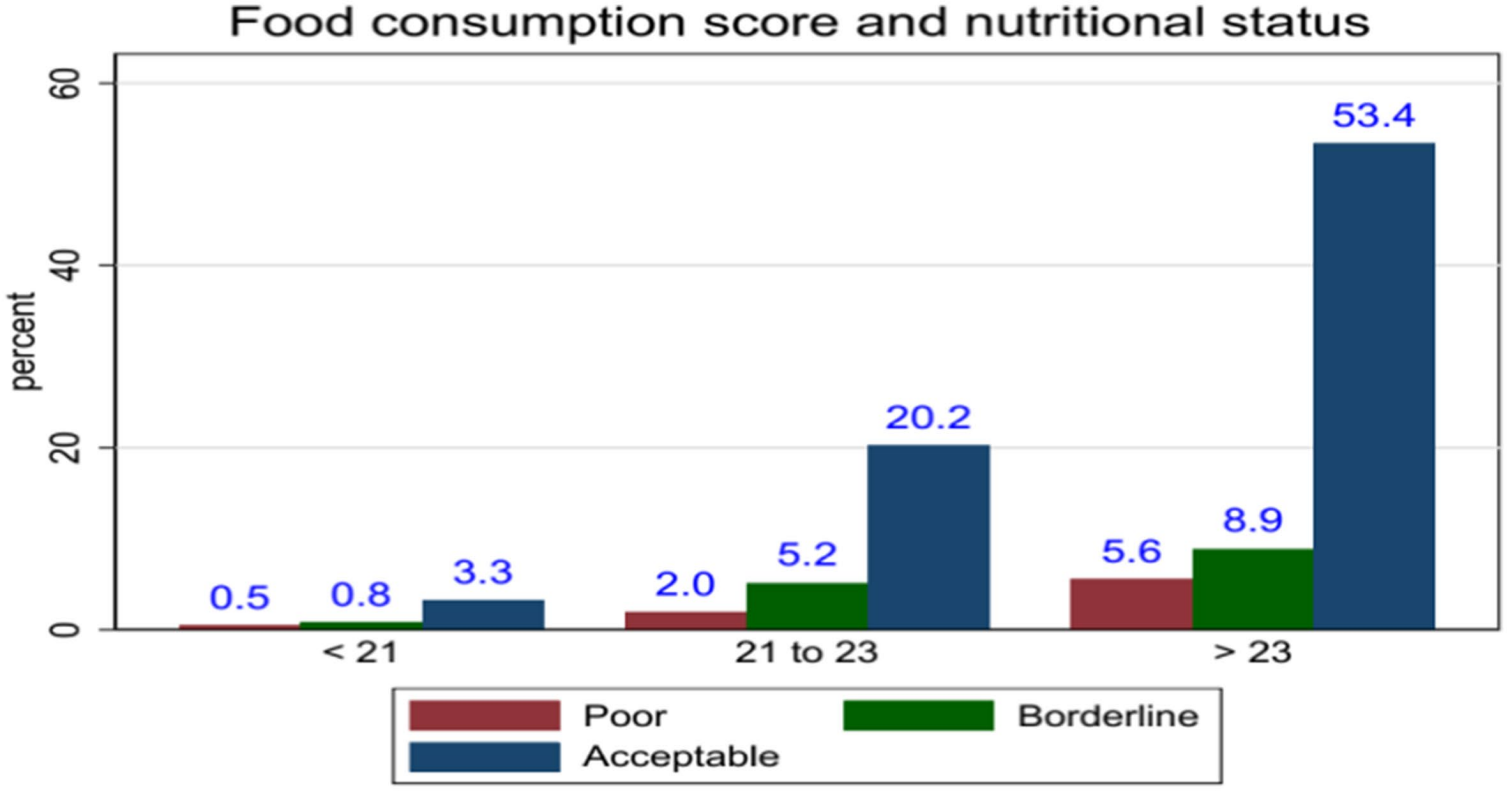
Magnitude of food consumption score and nutritional status among pregnant women in AM-HDSS, Gamo Zone of South Ethiopia.

The food consumption score among pregnant women showed that 76.96% had an acceptable, 14.89% had a borderline, and 8.14% had a poor. Regarding nutritional status, 68.03% had MUAC > = 23 cm, 27.43% had MUAC between 21 and 23 cm, and 4.55% had MUAC less than 21 cm (Figure 1).

Among the participants, 59.72 and 53.61% achieved high dietary diversity and food variety scores, respectively. About two-thirds 63.32% of the pregnant women had a meal frequency of four or more times per day. The majority 88.24 and 83.39% of the respondents consumed coffee leaf tea beverages and did not receive information about dietary intake during pregnancy (Table 2).

**Table 2:** Dietary related characteristics of the study participants in AM-HDSS, Gamo Zone of South Ethiopia.

Variable	Category	Number	Percent
Maternal minimum dietary diversity	Low	257	40.28
	High	381	59.72
	Mean (± SD)	4.74 (± 1.76)	
Food Varity score	Low	296	46.39
	High	342	53.61
	Mean (± SD)	55.34 (± 19.83)	
Meal frequency	≤ 3	234	36.68
	> 3	404	63.32
	Mean (± SD)	3.66 (± 0.61)	
Alcohol consumption	Yes	21	3.29
	No	617	96.71
Drink coffee	Yes	302	47.34
	No	336	52.66
Drink coffee leaf tea	Yes	563	88.24
	No	75	11.76
None nutritional crave	Yes	40	6.27
	No	598	93.73
Food aversion	Yes	91	14.26
	No	547	85.74
Receive dietary counseling	Yes	106	16.61
	No	532	83.39
Iron folic acid started	Yes	391	61.29
	No	247	38.71
Maternal nutritional knowledge	Poor	298	46.71
	Moderate	270	42.32
	Good	70	10.97
MUAC	Mean (± SD)	23.68 (± 2.23)	
BMI	Mean (± SD)	23.36 (± 2.55)	

### Determinants of food consumption score

The level of acceptable food consumption score increased by 2, 3, and 4% for every one-unit increase in BMI (APR = 1.02; 95% CI: 1.01, 1.04), MUAC in cm (APR = 1.03; 95% CI: 1.01, 1.05), and gestational age in weeks (APR = 1.04; 95% CI: 1.01, 1.07), respectively. Food consumption score was higher among women who were urban dwellers (APR = 1.09; 95% CI: 1.02, 1.20), in higher economic status (APR = 1.05; 95% CI: 1.01, 1.16), had planned pregnancy (APR = 1.13; 95% CI: 1.02, 1.25), were exposed to media (APR = 1.19; 95% CI: 1.07, 1.31), had a vegetable garden (APR = 1.14; 95% CI: 1.04, 1.25), had ANC visit (APR = 1.13; 95% CI: 1.02, 1.26), and those who consumed food four or more times per day (APR = 1.23; 95% CI: 1.11, 1.36) (Table 3).

**Table 3:** Factors associated with food consumption score among pregnant women in AM-HDSS, Gamo Zone of South Ethiopia.

Variable	Category	Food consumption score		CPR (95% CI)	APR (95% CI)
		Acceptable	Unacceptable		
Place of residence	Rural	435 (88.59)	141 (95.92)	Ref	Ref
	Urban	56 (11.41)	6 (4.08)	1.20 (1.09, 1.31)	1.09 (1.02, 1.20)
Educational status	No formal education	166 (33.81)	71 (48.30)	Ref	
	Formal education	325 (66.19)	76 (51.70)	1.16 (1.05, 1.27)	
Partner educational status	No formal education	197 (40.12)	76 (51.70)	Ref	
	Formal education	294 (59.88)	71 (48.30)	1.12 (1.02, 1.22)	
Households asset decision-making	By both partner	101 (20.57)	17 (11.56)	1.14 (1.04, 1.25)	
	Mainly by husband	390 (79.43)	130 (88.44)	Ref	
Wealth status	Low	164 (33.40)	54 (36.73)	Ref	Ref
	Middle	151 (30.75)	57 (38.78)	0.96 (0.86, 1.08)	0.98 (0.87, 1.10)
	High	176 (35.85)	36 (24.49)	1.11 (1.01, 1.22)	1.05 (1.01, 1.16)
Maternal nutritional knowledge	Poor	235 (47.86)	63 (42.86)	Ref	
	Moderate	204 (41.55)	66 (44.90)	0.96 (0 0.88,1.05)	
	Good	52 (10.59)	18 (12.24)	0.94 (0.81, 1.09)	
Current pregnancy	Planned	352 (71.69)	81 (55.10)	1.20 (1.08, 1.33)	1.13 (1.02, 1.25)
	Unplanned	139 (28.31)	66 (44.90)	Ref	Ref
Mass media exposure	Yes	356 (72.51)	76 (51.70)	1.26 (1.13, 1.40)	1.19 (1.07, 1.31)
	No	135 (27.49)	71 (48.30)	Ref	Ref
Had vegetable garden	Yes	333 (67.82)	76 (51.70)	1.18 (1.07, 1.30)	1.14 (1.04, 1.25)
	No	158 (32.18)	71 (48.30)	Ref	Ref
ANC visit	Yes	356 (72.51)	77 (52.38)	1.23 (1.12, 1.39)	1.13 (1.02, 1.26)
	No	135 (27.49)	70 (47.62)	Ref	Ref
Meal frequency	≤ 3	154 (31.36)	80 (54.42)	Ref	Ref
	>3	337 (68.64)	67 (45.58)	1.27 (1.14, 1.40)	1.23 (1.11, 1.36)
Receive dietary counseling	Yes	91 (18.53)	15 (10.20)	1.14 (1.04, 1.25)	
	No	400 (81.47)	132 (89.80)	Ref	
Gestational age (weeks)	Mean (± SD)	18.27(+ 1.69)	17.81(+ 1.77)	1.04 (1.01, 1.06)	1.04 (1.01, 1.07)
Body mass index	Mean (± SD)	23.61 (± 2.64)	22.51 (± 2.01)	1.04 (1.02, 1.05)	1.02 (1.01, 1.04)
MUAC	Mean (± SD)	23.88 (+ 2.20)	23.07 (+ 2.21)	1.04 (1.02, 1.06)	1.03 (1.01, 1.05)

## Discussion

Nutrition is crucial for the proper functioning of the body systems. However, chronic energy deficiency is common among pregnant women due to consuming unbalanced diets lacking adequate nutrients (42). This deficiency is exacerbated by food taboos, infectious diseases, food insecurity, and poor dietary knowledge, leading to a higher risk of adverse birth outcomes, morbidity, and mortality (43). This study aims to determine the level of food consumption score and associated factors among pregnant women in Gamo Zone, South Ethiopia.

The current study report revealed that about three-fourths (76.96, 95% CI: 73.52–80.07%) of the study participants had acceptable FCS. In contrast, less than two out of ten participants had borderline (14.89%) or poor (8.15%) FCS. This finding is consistent with the results from studies conducted in Nigeria (80.30%), and Northwest Ethiopia (80.30 and 81.50%) (31, 44, 45), though it falls short of the WFP recommended threshold of 90% (24). However, it surpasses the findings from studies in Addis Ababa, Ethiopia (51.2%), Eastern Ethiopia (54.46%), and Bangladesh (58%) (30, 32, 46). This is due to the fact that dietary intake in Ethiopia is highly influenced by seasonal variation. During the autumn and winter seasons, food access, including fresh produce and staple foods, increases due to harvest time. Consequently, the FCS also increases (31, 32). In contrast, the FCS decreases during fasting months when consumption of animal source foods is restricted due to religious rules (32, 47). Also the difference is probably due to cultural geographical and religious difference among the studies areas.

A total of 204 pregnant women (31.97, 95% CI: 28.46–35.70%) of the respondents were undernourished, with a MUAC measurement of less than 23 cm. This finding was nearly comparable to the pooled prevalence of under nutrition among pregnant women in Ethiopia (32%) (48), and the Afar regional state of Ethiopia (30.9%) (49). However, it was lower than studies from Jordan (49.2%) (50), western Ethiopia (39.2%) (51), southern Ethiopia (38 to 44.90%) (52, 53), eastern Ethiopia (43.8 to 47.90%) (54, 55), and southwest Ethiopia (42.4%) (56). It was also higher than recent studies conducted in various parts of Ethiopia (17.7, 21, and 27.6%) (57–59). The discrepancies in the findings may arise from variations in the timing of enrollment. In this study, participants were enrolled during early pregnancy, a period commonly associated with nausea and vomiting, which can result in reduced body fluid and subsequently lower MUAC measurements. Also the discrepancies could be attributed to differences in study settings, sociocultural and socioeconomic factors, sample sizes, and variations in the MUAC cutoff points.

In this study, the nutritional status of pregnant women showed a direct correlation with their food consumption score. Specifically, each 1 kg/m^2^ increase in BMI was associated with a 1.02 times higher in level of acceptable food consumption score, and each 1 cm increase in MUAC was linked to a 1.03 times higher level of acceptable food consumption score. This is in line with findings from earlier studies conducted across different regions of Ethiopia, indicating that food insecurity increases the risk of under nutrition among pregnant women (48, 51, 52, 56). Therefore, the nutrition program should focus on promoting healthy weight gain, regular monitoring of BMI and MUAC, and providing supplementation for pregnant women with low BMI or MUAC, ultimately enhancing their nutritional status and improving pregnancy outcomes.

In low-and middle-income countries like Ethiopia, limited accessibility to healthcare and underutilization of maternal health services among rural women prevents them from receiving adequate awareness about healthy dietary practices during pregnancy (60), resulting in lower food consumption scores. Furthermore, prevalent food taboos among rural pregnant women limit their intake of specific foods, contributing to reduced food consumption scores (14). The present study corroborates this, where the level of acceptable food consumption score was 1.09 times higher among urban dwellers compared to their counterpart. This finding was supported by a previous study conducted in eastern Ethiopia, indicating that urban pregnant women had a higher consumption of five or more food groups (61). In contrast, research from northwest Ethiopia showed that rural women had a higher FCS (31). This discrepancy is likely due to differences in the study setting and participants. The current study was community-based, with a majority of participants from rural areas, whereas the former study was hospital-based, with most participants from urban areas.

In this study, pregnant women from higher wealth status had a higher level of acceptable food consumption score than those from lower wealth status. It is a known fact that wealth plays a crucial role in meeting basic human needs, accessing healthcare services, and ensuring adequate dietary intake in terms of both frequency and quality (62). Consequently, individuals with lower socio-economic status often face lower food consumption scores due to financial constraints limiting their ability to purchase sufficient food. Similarly a previous study conducted in Ethiopia has shown that financial limitations and household income are frequently quoted as barriers to dietary intake during pregnancy (63). The finding of this study is consistent with studies conducted in Ethiopia (30, 32). However, wealth status is not associated with acceptable food consumption scores among pregnant women in Northwest Ethiopia (31). Therefore, addressing access to affordable and nutritious food through food assistance, subsidized food packages, or the special Productive Safety Net Programme for poorer pregnant women would be helpful.

The level of acceptable food consumption score was 1.13 times higher among participants who had planned pregnancies compared to those with unplanned pregnancies. This may be attributed to a higher incidence of maternal mental disorders like depression, anxiety, and stress among women with unplanned pregnancies (64), as well as lower utilization of maternal health services (65), which reduces the level of food consumption score. A study conducted in Iran supports this finding by reporting an association between unplanned pregnancies and food insecurity (66). Additionally, it is consistent with evidence from low-and middle-income countries, which suggests that unwanted pregnancies can affect maternal nutrition (65). Similarly pregnant women who were exposed to audiovisual mass media had a higher acceptable food consumption score compared to those who were not exposed. This finding was supported by studies done in Bangladesh, Indonesia and Ethiopia, where it was observed that mothers exposed to print and audiovisual media tended to offer a wider variety of foods to their infants (67–69). This is probably due to audiovisual information improvements awareness, attitudes, and practices regarding healthy behavior. Moreover, women who are exposed to media are more likely to obtain dietary information, which can lead to a higher food consumption score.

This study found that the acceptable food consumption score was 1.13 times higher among participants who visited a healthcare facility for antenatal care compared to those who did not visit. This is consistent with a study conducted in Western and Eastern Ethiopia (30, 70). This may be due to the increased likelihood of receiving information about the importance of dietary diversity and increasing meal frequency during pregnancy (71). However, ANC visits are not associated with the FCS among pregnant women in Addis Ababa, Ethiopia (32). This discrepancy is likely due to differences in study settings: the current study was community-based and primarily involved rural residents, while the previous study was facility-based and on urban residents. Similarly, the level of FCS was 1.04 times higher for every one-week increase in gestational age. This is similar with the study conducted in Western Ethiopia (70). This is the fact that, as gestational age increases, morning sickness tends to lessen, leading to an increase in dietary intake (72).

This study found that the likelihood of having an acceptable food consumption score was 1.23 times higher among pregnant women who consumed four or more meals per day compared to those who ate at least three meals per day. This might be due to the fact that increased meal frequency enhances dietary diversity, which in turn raises the food consumption score. This finding was supported by evidence from Western and Eastern Ethiopia, where meal frequency had a significant effect on food consumption (61, 70, 73, 74). Similarly, the level of FCS was higher among participants from households with home gardening compared to those without home gardening. This might be due to the prolonged increase in availability of different food groups in households with home gardening (75). This finding is in line with studies conducted in Eastern and Northwest Ethiopia, which found that the consumption of minimum dietary diversity is higher among pregnant women from households with home gardening (69, 73). Therefore, enhancing meal frequency counseling, providing training, and offering resources to support home gardening, which enables households to grow nutritious food, can help improve dietary practices during pregnancy.

### Strength and limitation of the study

In this study, using both the 24-h recall method and a one-week dietary history, along with conducting the assessment at the community level enhances the quality and accuracy of the data. Despite employing rigorous measurement, there is a chance that respondents over-reported or under-reported their dietary intake due to social desirability and recall bias. Additionally, as this is a cross-sectional study, it cannot establish causality.

## Conclusion

The level of acceptable food consumption scores was low in the study area, and under nutrition was high among pregnant women. This study identified several factors independently associated with the level of food consumption scores among pregnant women, including urban residence, higher wealth status, planned pregnancy, exposure to audiovisual mass media, having a vegetable garden, visiting health facilities for antenatal care, and consuming four or more meals per day. Additionally, food consumption scores were higher with increasing gestational age, body mass index, and mid-upper arm circumference. Thus addressing these factors is crucial for improving FCS among pregnant women. Furthermore, efforts should be directed toward increasing media exposure, boosting household income, promoting vegetable gardening, and improving planned pregnancies and antenatal care visits.

## Data Availability

The original contributions presented in the study are included in the article/supplementary material, further inquiries can be directed to the corresponding author.

## Glossary

AIC: Akaike’s Information Criterion
AM-HDSS: Arba Minch Health and Demographic Surveillance site
ANC: Anti Natal Care
APR: Adjusted Prevalence Ratio
ASFC: Animal Source Food Consumption
BIC: Bayesian Information Criterion
BMI: Body Mass Index
EDHS: Ethiopian Demographic and Health Survey
FCS: Food Consumption Score
FFQ: Food Frequency Questionnaire
FVS: Food Variety Score
HIV: Human Immunodeficiency Virus
IRB: Research Ethics Review Board
IUGR: Intrauterine Growth Restriction
LBW: Low Birth Weight
MDDW: Minimum Dietary Diversity for Women
MUAC: Mid Upper Arm Circumference
PCA: Principal Component Analysis
VIF: Variance Inflation Coefficients
WFP: World Food Programme
WHO: World Health Organization
